# Self-Reports of Past Malevolent Behavior and Narcissism as Predictors of Malevolent Creativity on a Real-World Ideation Task

**DOI:** 10.3390/jintelligence14080176

**Published:** 2026-08-01

**Authors:** Natalie A. Ceballos, Ruby N. Sweet, Carmen E. Westerberg

**Affiliations:** Department of Psychology, Texas State University, San Marcos, TX 78666, USAcw54@txstate.edu (C.E.W.)

**Keywords:** AMORAL model, dark triad, divergent thinking, idea generation, malevolent creativity, problem solving

## Abstract

Creativity has a dark side—malevolent creativity (MC), which is the application of original ideas to purposely harm others; i.e., creative problem-solving with malicious intent. Here, the influence of past MC behavior on MC ideation was examined while controlling for other individual-level factors relevant to the AMORAL model of MC. College undergraduates (*N* = 276 initially recruited; *N* = 248 final sample analyzed; 82% female) were surveyed on demographics, self-perception of problem-solving potential (SP-PSP), dark triad traits, and past MC behavior, and completed an adapted Malevolent Creativity Task (MCT), generating revenge ideas to an unfair scenario. Sequential regression models were used to predict MCT outcomes (fluency index, malevolence index, and composite score), incrementally controlling for age, sex, SP-PSP, dark triad traits, past MC behavior, and provocation level. In the final model, SP-PSP significantly predicted the MCT fluency index (*R*^2^ = 0.065). Past MC behavior significantly increased the *R*^2^ of the MCT malevolence index; the final model was significant (*R*^2^ = 0.084). Past MC behavior and narcissism significantly predicted the MCT composite score (*R*^2^ = 0.091). These findings align with and extend previous research linking past MC behavior to MC ideation by highlighting unique and incremental impacts of SP-PSP, narcissism, past MC behavior, and provocation on MC ideation.

## 1. Introduction

Creativity is typically perceived to have a positive impact on society. Psychologists define creativity as requiring both originality and effectiveness, and a recent update to that definition also emphasizes the authenticity and intentionality of human creativity versus artificial intelligence (Barron, 1955; Plucker et al., 2004; Runco & Jaeger, 2012; Runco, 2025). However, as Kapoor and Kaufman (2022a) point out, criminals’ creative solutions can also be both original and effective in helping them to achieve antisocial ends. Researchers alluded to this phenomenon as early as the 1990s, calling it “dark” creativity (McLaren, 1993) or “negative” creativity (James et al., 1999; Clark & James, 1999). Later, D. H. Cropley et al. (2008) explicitly framed malevolent creativity as a distinct concept defined by originality, effectiveness, and intent, specifying that malevolent creativity involves deliberately harming others. Today, malevolent creativity is typically defined as the application of original ideas to purposely harm others and/or gain an advantage through actions such as manipulation, threat, or harm; it is creative problem-solving with malicious intent (A. J. Cropley, 2010; D. H. Cropley, 2010). Elements of malevolent creativity are evident in more mundane transgressions like malicious pranks, as well as in criminal activity and large-scale violence like terrorism (Chang, 2026; A. Cropley & Cropley, 2011; D. H. Cropley & Cropley, 2013; Eisenman, 2008). Runco argues that creativity itself is neither good nor bad; rather, it is a tool that may be deployed for positive or negative purposes (Runco, 2010). In this way, the “dark side” of creativity is merely a function of values and decisions unrelated to the creative processes themselves (Runco, 2010).

### 1.1. The AMORAL Model

Kapoor and Kaufman’s (2022a, 2023) AMORAL model (Antecedents, Mechanisms, Operants, Realization, Aftereffects, and Legacy) provides an interdisciplinary framework for how and why dark, negative, and malevolent types of creativity may materialize. The AMORAL model includes a very large number of factors that may influence the pathway to malevolently creative behavior, as well as the aftereffects and legacies of such actions. These include antecedents (i.e., the “A” in AMORAL; such as drivers, motivations, and belief systems), individual-level mechanisms (i.e., the “M” in AMORAL; such as personality and the propensity to be creative), and environment-level operants (i.e., the “O” in AMORAL; including contextual cues, situational triggers, and social and material assets that may expand or constrain the development of a malevolent idea), all of which may act together to set the stage for the potential realization, aftereffects, and legacies (i.e., the “RAL” in AMORAL) of creatively malevolent activity.

#### 1.1.1. Antecedents (The “A” in AMORAL)

The antecedent layer of the AMORAL model represents the core drivers of malevolent creativity, including power, resources, hedonism, and belief systems. In the context of malevolent creativity, these motivators are turned toward selfish or self-serving ends. Kapoor and Kaufman (2022a) provide real-world examples. An office worker with a need for power might undermine their colleagues to get a promotion. A CEO striving to obtain more resources might engage in corporate corruption. In the pursuit of pleasure, a hedonistic thinker might engage in immoral recreational activities. In an attempt to advance their belief system, an ideological extremist might commit a large-scale act of violence. While antecedents are foundational factors that may drive malevolent creativity, they can be challenging to manipulate within a research setting. The current study focused primarily on the “M” and the “O” of the AMORAL model.

#### 1.1.2. Individual-Level Mechanisms (The “M” in AMORAL)

According to Kapoor and Kaufman (2022a), if antecedents are drivers, then individual-level mechanisms may act as an accelerator, or a handbrake on the forward motion of malevolent creativity toward antisocial ends. These individual-level factors include intellectual ability, the propensity to be creative, personality, personal values, socioemotional skills, and action-relevant knowledge (e.g., possessing the specific skills to execute a malevolent plan). Of these, the propensity for creativity, personality, and action-relevant knowledge are most relevant to the current study. Kapoor and Kaufman (2022a) note that their inclusion of the individual factor of “propensity for creativity” distinguishes the AMORAL framework as a model for malevolent creativity rather than merely malicious behavior. It is interesting to note that Vincent and Goncalo (2014) suggest that merely perceiving oneself to be creative is sufficient to motivate the malevolent behavior of dishonesty with or without actual creative talent.

In terms of personality, the dark triad features (Machiavellianism, narcissism, and psychopathy) are particularly relevant to malevolent creativity. While the dark triad personalities share a callous, manipulative core, each trait also exhibits distinct characteristics (Paulhus & Williams, 2002; Paulhus, 2014). Individuals with Machiavellian traits tend to be strategic, manipulative, and indifferent to morality (Bereczkei, 2017; Jones, 2016). Narcissists, who are characterized by egocentrism, grandiosity and the need for admiration, tend to have exaggerated responses to perceived slights (Dowgwillo et al., 2016; Rasmussen, 2016). Psychopaths lack empathy and tend to act impulsively (Hare, 1999), which may manifest in a dysfunctional form associated with poor planning (i.e., “look before you leap”) or a more functional form (i.e., “seize the moment”) as seen in criminal behavior that requires a higher degree of planning and persistence (Ben-Yaacov & Glicksohn, 2020; Smillie & Jackson, 2006, p. 48; Weidacker et al., 2017) and in “successful psychopaths” who evade punishment (Y. Gao & Raine, 2010).

In their comprehensive review and meta-analysis of the literature on general creativity and the dark triad, Lebuda et al. (2021) note that, since the 1980s (e.g., Raskin, 1980; Solomon, 1985), narcissism has been the dark triad trait that is most frequently linked to general creativity, with varying strengths of association depending on how creativity is operationalized. Typically, positive associations have been noted between narcissism and self-report measures of creativity, including measures of self-perceived creative potential (Dahmen-Wassenberg et al., 2016; Hughes et al., 2013; Goncalo et al., 2010; Jonason et al., 2015; Lebuda et al., 2021; McKay et al., 2017). However, in studies that use independent evaluation or blind coding of participants’ creativity, the relationships between narcissism and creativity are weaker or non-significant, suggesting that narcissists may perceive themselves to be more creative than they actually are (Goncalo et al., 2010; Lebuda et al., 2021; Sordia et al., 2022). The associations between general creativity and the other dark triad traits (i.e., Machiavellianism and psychopathy) tend to be less compelling. Previous studies have shown that Machiavellian individuals exhibit lower fluency and originality in divergent thinking tasks compared to non-Machiavellians, but Machiavellians also tend to generate more ideas that explicitly involve harm to others (Dahmen-Wassenberg et al., 2016; Jonason et al., 2017; Kapoor, 2015; Lebuda et al., 2021). Lebuda et al.’s (2021) meta-analysis found statistically significant associations between general creativity and Machiavellianism, though this link was not as strong as the relationship between general creativity and narcissism. Their meta-analysis also found that the relationship between psychopathy and general creativity was not significant when all types of creativity were considered as a whole; further, given that psychopaths tend to be more interested in realistic and practical activities, it is interesting to note that links between everyday creativity and psychopathy were also non-significant (Galang et al., 2016; Jonason et al., 2014; Lebuda et al., 2021).

With their common characteristics of selfishness and manipulation, it seems intuitive that individuals with dark triad traits might be more likely to use their creativity to cause harm to others, and in fact, numerous studies have linked the dark triad to dark, negative, or malevolent creativity (Z. Gao et al., 2022; Jia et al., 2020; Kapoor & Kaufman, 2022b; Szabó et al., 2022). Most recently, Silva et al. (2025) found that malevolent creativity positively mediated the relationship between all three dark triad traits and negative deviant behavior. Dow (2023) notes that while Machiavellianism, narcissism, and psychopathy are all linked to malevolent creativity, these different personality styles may express malevolent creativity in different ways and in response to different situations. Specifically, while Machiavellians may be more proactive and goal-oriented, psychopaths tend to be reactive in their responses to physical provocation, and narcissists are more likely to respond aggressively to ego threat provocations, such as personal insults (Jones & Paulhus, 2010; Paulhus et al., 2018).

Finally, a person’s life experiences, including their past malevolently creative behaviors and the outcomes of those behaviors, contribute to their action-relevant knowledge. Perchtold-Stefan et al. (2025) have described a similar concept in the creation of an individual’s “aggressive expertise,” which may shape the way that they perceive and respond to their world. In this way, past malevolently creative behaviors may align with the individual-level factor of the AMORAL model, acting as an accelerator, or a handbrake on the forward motion of malevolent creativity toward antisocial ends.

#### 1.1.3. Environment-Level Operants (The “O” in AMORAL)

The next layer of the AMORAL model situates individuals within the larger environment, which is filled with potential amplifiers, modifiers, and attenuators of malevolent creativity. According to Kapoor and Kaufman (2022a), these environment-level factors may include cultural ideology and the material and social assets that might be available for use in malevolent plans. Importantly, the AMORAL model acknowledges that malevolent creativity is sometimes reactive (rather than proactive) and may be provoked by contextual factors and situational triggers, particularly in individuals with high trait anger or aggression (Cheng et al., 2021; Gutworth et al., 2018; Harris & Reiter-Palmon, 2015; Lee & Dow, 2011; Perchtold-Stefan et al., 2021b; Zhang et al., 2024).

Different types of provocation have been studied in relation to malevolent creativity. Perceived unfairness and social exclusion may specifically prompt susceptible individuals to weaponize their environmental affordances in malevolently creative ways (Baas et al., 2019; Li et al., 2025; Wu et al., 2025; Zhang et al., 2024). For more reactive individuals with lower levels of inhibitory control, this weaponization might take the form of immediate violence (e.g., using a heavy textbook as an improvised bludgeon; Dumas & Strickland, 2018; Perchtold-Stefan et al., 2021a). However, for others, malevolent creativity may be applied to construct long-term revenge plots, involving delayed gratification, cognitive flexibility, and stage-based creative modeling, in which individuals actively generate multiple ideas, evaluate their effectiveness, and select the most damaging approach (Mumford et al., 1991; Perchtold-Stefan et al., 2025; Perry-Smith & Mannucci, 2017; Rietzschel & Ritter, 2018; Wang et al., 2025). Perchtold-Stefan et al. (2025) note that an individual’s perception of the meaning of a provoking incident may also be shaped by their life experiences and “aggressive expertise,” potentially influencing the problem construction phase of creative problem solving (e.g., Reiter-Palmon et al., 2024) and leading to the justification of more harmful creative solutions. The capacity for “aggressive expertise” to influence malevolent creativity may also align with the AMORAL model’s individual-level mechanism of “action-relevant knowledge” (Kapoor & Kaufman, 2022a).

#### 1.1.4. Realization, After Effects, and Legacies (The “RAL” in AMORAL)

Within the AMORAL framework, research that uses real-world scenarios to provoke the generation of hypothetical ideas for malevolently creative actions (e.g., Harris & Reiter-Palmon, 2015; Perchtold-Stefan et al., 2021a) may fall under the realization component of the model (i.e., the R in AMORAL), specifically dealing with the nature of the target and the continuum of proximity; that is, the gap between original thought and its behavioral counterpart, a corresponding action. In terms of the translation of malevolently creative ideas into malevolent actions, others have described this process as malevolent innovation (d’Amato & Hunter, 2025; Hunter et al., 2022), occurring in a three-stage process of malevolent idea generation, championing, and implementation (Wang et al., 2025). Aftereffects and legacies are the final two stages of the model (i.e., the “AL” in AMORAL), which refer to the fallout of a malevolently creative action over the short and long term, respectively (Kapoor & Kaufman, 2022a, 2023). While a person’s past malevolently creative behaviors are relevant to earlier layers of the AMORAL model (e.g., individual-level factors like action-relevant knowledge), past behavior might also align with aspects of realization, aftereffects, and legacies in that recollection of past behavior describes actual instances in which malevolent ideation led to malevolent actions and their consequences.

### 1.2. Measuring Creativity

General creativity is measured using a diverse set of tasks and/or questions focused on different aspects of creativity. Simple questionnaires can be used to assess creativity based on past achievements (Carson et al., 2005), frequency of engagement in creative endeavors (Ivcevic, 2007), or frequency of creative ideation (An et al., 2016; Runco et al., 2001). The Kaufman Domains of Creativity Scale (K-DOCS) measures perceived creative abilities in specific domains (e.g., writing, science, performance, art) in addition to everyday creativity (Kaufman, 2012). Other questionnaires assess personality traits frequently associated with creativity (e.g., intuition; Gough, 1979). Cognitive psychologists typically distinguish between divergent and convergent thinking, where divergent thinking refers to the ability to generate multiple, original and diverse ideas, whereas convergent thinking involves selecting the best or most appropriate solution to a problem among a set of alternatives (Jaarsveld et al., 2012). In convergent thinking tasks, divergent thinking may be involved in generating possible solutions, but the usefulness of these solutions must be evaluated to generate a single answer (Brophy, 2001; A. Cropley, 2006).

Divergent thinking is frequently considered a proxy for creativity (e.g., Paek et al., 2021; Reiter-Palmon et al., 2019; Runco & Acar, 2012), while less attention has been given to the role of convergent thinking in creativity (Rawlings et al., 2025). A classic test of divergent thinking is the Alternative Uses Task (Guilford, 1967), in which participants are asked to generate as many possible uses as they can for a common, everyday object (e.g., a brick), and then responses are scored based on frequency, originality (uniqueness), flexibility (number of different categories of uses), and elaboration (amount of detail given). The Torrance Tests of Creative Thinking assess divergent thinking in a similar manner (Torrance, 1966; Runco et al., 2010). Performance on these tasks is hypothesized to depend on the breadth of associative networks, such that having more and broader associations between concepts (i.e., more links between weakly associated concepts) leads to increased divergent thinking (Mednick, 1962). Others argue that analogical thinking breeds creativity (Weisberg, 1995). In analogical thinking, a conceptual structure from one context is applied to a new, innovative context. Once the mapping has been made from one context to another, additional features of the structure in the new context may become apparent (Dunbar, 1995). Analogical thinking has been associated with creativity in multiple domains (Dunbar, 1997; Gentner & Colhoun, 2010; Welling, 2007).

Divergent, analogical thinking (i.e., general creativity) undoubtedly plays a role in malevolent creativity, as both general and malevolent creativity involve the generation of novel ideas based on associative and flexible thinking. Recent research has found positive relationships between general and malevolent creativity abilities (e.g., Dumas & Strickland, 2018; Perchtold-Stefan et al., 2021a; Xu et al., 2023), and similar brain activity has been observed while individuals are engaging in general compared with malevolent creativity (Z. Gao et al., 2025), supporting the notion of shared cognitive processes between general and malevolent creativity. However, general creativity is most strongly predictive of malevolently creative fluency (i.e., quantity of ideas generated) but is not related to other affective, personality, and environmental factors known to influence malevolent creativity (Perchtold-Stefan et al., 2021a), indicating additional mechanisms must contribute.

### 1.3. The Challenges of Measuring Malevolent Creativity

For a variety of reasons, it is difficult to measure malevolent creativity via self-report. First, as noted by Szabó et al. (2022), malevolently creative behavior can take many forms, and measuring all of them in a questionnaire presents a psychometric challenge. Second, retrospective bias and social desirability effects may hinder reporting of malevolently creative behavior, particularly among those who may wish to appear prosocial. Finally, in terms of conceptual accuracy, it can be difficult to capture the intersection of malevolence and creativity through a subjective scale; more objective measures are needed, such as the generation of malevolently creative ideas (Perchtold-Stefan et al., 2025). To that end, researchers have measured malevolent variants of divergent thinking by measuring the number of unsolicited, malevolent ideas generated by traditional alternate uses tasks (e.g., weaponizing a brick), as well as the proportion of total ideas that were judged to be malevolent (e.g., Lee & Dow, 2011; Dumas & Strickland, 2018). Others have used tasks designed to elicit malevolently creative ideas in response to problems in which participants imagine being treated unfairly (Harris & Reiter-Palmon, 2015), as well as timed malevolent creativity tasks with a similar focus on revenge scenarios (Perchtold-Stefan et al., 2021a). Problem or scenario-based approaches have been used in college student samples from various cultures (e.g., in Austria, China, and the Midwestern United States) (e.g., Harris & Reiter-Palmon, 2015; Hao et al., 2020; Perchtold-Stefan et al., 2021a, 2021b) and in studies of participants under correctional supervision (e.g., Eisenman, 2008; Perchtold-Stefan et al., 2024; Szabó et al., 2022). In their study of Romanian prisoners, Szabó et al. (2022) found that more experienced criminals exhibited higher levels of malevolently creative ideation, which may suggest that past malevolent behavior facilitates current malevolently creative ideation (i.e., practice makes perfect). This could be realized through reinforcement learning (e.g., Thorndike, 1911), such that when malevolently creative behaviors are rewarded, they are more likely to occur again. With repeated rewards, malevolently creative ideation may become habitual and automatic (Graybiel, 2008). However, to date, the influence of past malevolent creativity on current malevolently creative ideation and the alignment of these factors within the AMORAL model have not been examined.

### 1.4. The Current Study

This cross-sectional study aimed to address a gap in the literature on malevolent creativity by determining whether past malevolently creative behavior influences malevolently creative ideation above and beyond other individual-level mechanisms (in the parlance of the AMORAL model) known to influence malevolently creative ideation, specifically the dark triad personality traits and general creative ability (e.g., Z. Gao et al., 2022; Jonason et al., 2017; Kapoor, 2015; Perchtold-Stefan et al., 2021a). Importantly, the study also determined whether current provocation level (aligning with the environment level in the AMORAL model) influences malevolently creative ideation beyond individual-level mechanisms and past malevolently creative behavior.

Past malevolently creative behavior was measured with a self-report questionnaire known as the malevolent creativity behavior scale (MCBS; Hao et al., 2016) that covered acts such as lying (e.g., How often do you fabricate lies to simplify a problem situation?), hurting people (e.g., How often do you think about ideas to take revenge when being unfairly treated?), and playing tricks (e.g., How often do you have ideas about how to pull pranks on others?). Malevolently creative idea generation was provoked in response to the Neighbor/Money scenario of the Malevolent Creativity Task (MCT; Perchtold-Stefan et al., 2021a), in which the participant is wronged by a neighbor in an apartment complex and asked to generate hypothetical ideas for revenge. Thus, although the survey was cross-sectional, it included a within-subject experimental element, in that participants were provoked by a stimulus (Neighbor/Money Scenario) and then generated ideas in real time, followed by a self-rating of their provocation level, which aligns as an environment-level factor in the AMORAL model. Previous studies have noted positive correlations between MCBS scores and the generation of malevolent ideas on the MCT and related tasks (Perchtold-Stefan et al., 2021a; Z. Gao et al., 2022; Hao et al., 2020). However, it is important to note that the semantic-level overlap between the two measures (e.g., recalling past instances of revenge behavior versus producing revenge ideas in real-time in response to a provoking scenario) could act to inflate their predictive relationship.

To address the individual-level mechanism of “propensity for creativity” from the AMORAL model, we measured self-perceived problem-solving ability using a single item adapted from the self/everyday creativity subscale of the K-DOCS (Kaufman, 2012). Participants were asked to rate their creative potential on “everyday creativity, such as choosing the best solution to a problem” using a scale of 1 (not creative at all) to 5 (very creative). This item has high face validity in that it is a concrete, unambiguous subjective measure of creativity in everyday problem-solving, addressing participants’ self-perceptions of “propensity for creativity”. While limited in a psychometric sense, the single-item approach (versus administering the scale) also decreases the threat of respondent fatigue.

Dark triad personality traits, which also align with the individual level of the AMORAL model, were measured using the Dark Triad Dirty Dozen Questionnaire (Jonason & Webster, 2010), which includes subscales for Machiavellianism, narcissism, and psychopathy.

Drawing on the framework of the AMORAL model and previous work on malevolently creative ideation (Harris & Reiter-Palmon, 2015; Perchtold-Stefan et al., 2021a, 2021b), we developed separate sequential linear regression models for each of the MCT outcomes (fluency index, malevolence index, and composite score, which incorporates originality as well as malevolence) to examine the incremental impact of specific theoretically-relevant variables or sets of variables while controlling for others, such that each block allowed us to observe changes in the variance explained as variables were added in stages. In block 1, age and sex were entered to control for potential age effects related to the frontal lobe development that occurs during the transition from late adolescence to adulthood, as well as potential sex differences in dark triad traits and/or malevolent creativity (e.g., Casey et al., 2008; Chiorri et al., 2019; Götz et al., 2020; Grijalva et al., 2015; Harris & Reiter-Palmon, 2015; Lee & Dow, 2011). Self-perception of problem-solving potential (SP-PSP) and dark triad personality traits (Machiavellianism, psychopathy, and narcissism) were entered in block 2. To determine if past malevolently creative (MC) behavior predicted MCT outcomes above and beyond SP-PSP and dark triad traits, past MC behavior was entered alone in block 3. Finally, to determine if current provocation level predicted MCT outcomes above and beyond individual-level factors and past MC behavior, provocation level was entered alone in block 4. It should be noted that because this study focuses on the contribution of only a small subset of factors posited to be relevant for MC behaviors (Kapoor & Kaufman, 2022a), the size of the hypothesized effects was expected to be relatively small. Of greatest interest was the relative contribution of past MC behaviors after accounting for the dark triad personality traits and SP-PSP. Our hypotheses were as follows:

**Hypothesis** **1.** *We predicted that SP-PSP (reflecting propensity for creativity, an individual-level predictor in the AMORAL model) would be positively related to all MCT outcome variables but would be most likely to predict the fluency index, which measures the total number of task-relevant items generated without prioritizing the level of malevolence or originality. Using a similar, single-item measure of self-perceived creativity, Wahbeh et al. (2024) found a significant positive correlation (with a small effect size) between self-perceived creativity and fluency on the AUT.*

**Hypothesis** **2.** *We predicted that dark triad traits (reflecting personality, an individual-level predictor in the AMORAL model) would be positively related to the MCT malevolence index and composite scores. Previous work by Z. Gao et al. (2022) supports this prediction. They reported that a combined measure of dark triad traits was positively associated with MCT performance with a small effect size. Given the tendency for narcissists to be more strongly reactive to social slights (Lambe et al., 2018), it is possible that narcissists might be more likely than the other dark triad types to malevolently ideate in response to the MCT Neighbor/Money scenario. In a similar study, Perchtold-Stefan et al. (2021a) found that antagonism (a core feature of narcissism) predicted MCT performance with a small to medium effect size. However, that study did not assess the dark triad traits directly; therefore, whether narcissism might contribute unique variance above and beyond the other dark triad traits remained an empirical question.*

**Hypothesis** **3.** *Past MC behaviors might align with the individual level (“M”) of the AMORAL model in terms of action-relevant knowledge that is built up over cycles of realization and aftereffects. An example of this might be the “aggressive expertise” described by Perchtold-Stefan et al. (2025), which could potentially accelerate the application of creativity toward malevolent ends. Accordingly, we predicted that MCBS scores (past MC behaviors) would be positively related to the MCT malevolence index and composite scores. Previous studies have found that these measures are correlated with small to medium effect sizes (Hao et al., 2020; Perchtold-Stefan et al., 2021a). We specifically predicted that MCBS scores would account for unique variance above and beyond that of the dark triad traits and SP-PSP. This prediction is supported by a similar study by Z. Gao et al. (2022), who found that as an individual predictor, MCBS scores predicted MCT performance with a medium effect size, within an overall model that accounted for general creative behavioral tendencies, dark triad traits, and trait aggression.*

**Hypothesis** **4.** *We predicted that provocation level (reflecting an environment-level factor in the AMORAL model) would be positively related to the MCT outcome scores but would be most likely to predict the malevolence index and composite scores with stronger provocation eliciting greater malevolent ideation. While Perchtold-Stefan et al. (2021a) did not examine provocation level as a predictor of MCT performance, they reported a mean provocation level of 4.37 on a scale of 0 = not provoked at all to 6 = very provoked and found that a conceptually related factor, state anger, was associated with the MCT composite score with a medium effect size. Whether current provocation level would predict MCT outcomes above and beyond the other factors (i.e., SP-PSP, dark triad traits, and past MC behaviors) remained an empirical question.*

If past MC behaviors uniquely predict MCT performance beyond SP-PSP and dark triad personality traits, this would provide additional support for the AMORAL model (Kapoor & Kaufman, 2022a) and provide new avenues for research to examine how additional factors beyond individual mechanisms contribute to malevolently creative ideation.

## 2. Materials and Methods

### 2.1. Study Design

This was a cross-sectional study in which participants completed an anonymous online survey administered via Qualtrics. First, participants responded to questions about demographics, past malevolently creative (MC) behaviors (MCBS scale), and self-perception of problem-solving potential (SP-PSP). Then, they completed a malevolently creative idea generation task in which they were provoked by a revenge scenario. At the end of the task, they rated how provoked they were by the scenario and then completed a dark triad personality questionnaire.

This study was approved by the participating university’s Institutional Review Board, in accordance with ethical standards of the Declaration of Helsinki. Participants provided written, informed consent prior to participation.

### 2.2. Participants

Between February and May of 2024, 276 undergraduates were recruited from multiple sections of an Introduction to Psychology course (a general core course option for all undergraduates) at a large university in the Southcentral United States. They were compensated with class credit through the university’s Human Subjects Pool. Participants were recruited as part of a larger, ongoing study of the psychology of creativity, which had no exclusionary criteria. To focus our work more specifically on demographically typical undergraduates, quantitative analyses included only male and female undergraduates between the ages of 18 and 25. Additional participants were excluded from statistical analyses for reasons detailed in Section 2.4.2. The final sample was composed of 248 participants.

### 2.3. Measures

#### 2.3.1. Demographics

Participants provided demographic information including age, sex (male or female), ethnicity (Hispanic or non-Hispanic), and race (White, Black or African American, Asian, Multiracial, or Other).

#### 2.3.2. Past Malevolently Creative (MC) Behaviors

Past malevolently creative behavior was measured using the Malevolent Creativity Behavior Scale (MCBS, Hao et al., 2016). The MCBS is a 13-item scale on which participants rate the frequency (0 = never to 5 = usually) of behaviors such as hurting people, lying, and playing tricks. An example item is “How often do you think about ideas to take revenge when being unfairly treated?”. For the current study, all items on the scale were averaged to represent a single, overall measure of the construct. Higher scores reflect a greater tendency toward malevolently creative behavior. In our participant sample, the overall measure had good internal consistency (Cronbach’s α = 0.86).

#### 2.3.3. Self-Perception of Problem-Solving Potential

For the current study, we assessed participants’ perceptions of their potential to engage in real-world creative problem-solving. This was measured with a single representative item adapted from the self/everyday domain of the Kaufman Domains of Creativity Scale (K-DOCS; Kaufman, 2012). Participants were asked to rate their creative potential on “everyday creativity, such as choosing the best solution to a problem” using a scale of 1 (not creative at all) to 5 (very creative). The K-DOCS subscale from which our single-item measure was derived has good internal consistency (Cronbach’s alpha = 0.86) and test-retest reliability (*r* = 0.80). We acknowledge that a single extracted item can have very different psychometric properties compared to a full subscale; however, the assessment of test-retest reliability and validity psychometrics for our single-item measure was beyond the scope of the current study. Similar single-item measures of self-perceived general creativity (Wahbeh et al., 2024) have shown good test-retest reliability (ICC = 0.79) and statistically significant positive relationships to both AUT fluency (*r* = 0.13) and originality (*r* = 0.11). However, because the use of a single-item measure could lead regression models to underestimate the true effect of self-perceived problem-solving potential, results pertaining to the SP-PSP should be further substantiated using traditional multi-item measures and objective tests of general creative ideation.

#### 2.3.4. Malevolent Creativity Task (MCT)

Next, participants completed the Neighbor/Money component of the Malevolent Creativity Task (MCT; Perchtold-Stefan et al., 2021a), which was embedded in the online survey. The Neighbor/Money scenario is a realistic, open-ended problem depicting unfair behavior from a peer/associate, which is designed to elicit at least moderate anger in the participants (Perchtold-Stefan et al., 2021a). The initial instructions were as follows: “Read the following scenario and then generate as many original ideas as possible to react to the unfair behavior depicted in the scenario, to get back at or sabotage the wrongdoer. Try to come up with original ways of revenge without your actions tracing back to you. This is a hypothetical situation, so you should feel free to produce ideas that are not typical for you or which you would not necessarily act out in daily life.” The Neighbor/Money scenario read as follows: “Your neighbor asks you to help them with renovations in their apartment and offers to pay you for your help. Since you are currently low on money, you agree. After the work is done, you ask them for the payment they promised. However, your neighbor insists that such an agreement never took place and that you just imagined the whole thing.” Participants were then reminded of their instructions: “In the space below, tell us all the ways that you could get revenge on your neighbor. Try to generate as many original ideas as possible. This is a hypothetical situation, so feel free to use ideas that you would not necessarily act out in real life.” Participants typed their responses in an open-ended manner without a time constraint. After participants had responded to the scenario, they were asked to “rate how provoked you would feel if this scenario happened to you in real life” using a scale of 0 = not provoked at all to 6 = very provoked.

#### 2.3.5. Dark Triad Personality Traits

To assess dark triad personality traits, we used the Dark Triad Dirty Dozen Questionnaire (Jonason & Webster, 2010). Participants indicated the extent of their agreement (1 = strongly disagree to 7 = strongly agree) with 12 statements concerning behaviors consistent with dark personality traits. Subscales included narcissism, psychopathy, and Machiavellianism. An example statement is “I tend to lack remorse” (psychopathy). Higher scores indicate greater endorsement of these personality traits. In our sample of participants, the subscales had good or acceptable internal consistency: Cronbach’s α = 0.74 (narcissism), 0.75 (psychopathy), and 0.79 (Machiavellianism).

### 2.4. Data Analysis

#### 2.4.1. MCT Scoring

MCT scoring was based on the procedure and rubric published by Perchtold-Stefan et al. (2021a). Scoring was conducted by two members of the research team: a senior faculty member with more than 20 years of psychological research experience and an upper-level undergraduate researcher with one year of training and experience in the coding of qualitative data. Raters first familiarized themselves with the MCT scoring rubric (Perchtold-Stefan et al., 2021a). Then, following Gutworth et al. (2018), the raters engaged in practice ratings of sample responses to identify and minimize biases, to ensure accurate and valid scores, and to identify potential contextual nuances relevant to the study population and geographical region of interest. Prior to the independent rating of actual study data, participants’ MCT data were coded with a participant number and separated from the rest of the study materials so that the raters were blind to participants’ demographics and responses to other study components. Because the MCT data were collected as part of a larger collaborative study on creativity, the raters scored the responses blind to specific hypotheses, despite being aware of the overarching study goals and existing research literature linking creativity and behavior. The raters scored independently and then compared their results to analyze inter-rater reliability.

The scoring process proceeded as follows. First, a fluency index was calculated as the total number of non-identical ideas generated in response to the MCT Neighbor/Money scenario that were task-relevant, having at least some element of malevolent intent (99.6% of all ideas). Then, each idea was rated for malevolence and originality by the two independent raters, and both inter-rater reliability (IRR; calculated as percent absolute agreement) and interclass correlation coefficient (ICC; two-way, consistency) were calculated. The malevolence of each idea was rated on a scale of 1 (slightly malevolent) to 4 (highly malevolent). Inter-rater reliability (IRR = 0.834) and the interclass correlation coefficient (ICC = 0.953) for malevolence ratings were very good. Next, the malevolence scores for the two raters were averaged for each idea. A malevolence index was calculated as the total number of non-identical ideas that were judged to be at least slightly malevolent using the aforementioned rubric (average malevolence rating ≥ 1). Although the fluency and malevolence indices are not entirely independent, computing them in this manner is consistent with prior studies using the MCT (e.g., Perchtold-Stefan et al., 2021a) and with evidence indicating overlapping but not identical neural activation patterns for general and malevolent creativity (Z. Gao et al., 2025). The same two raters judged the originality of the ideas on a scale of 1 (slightly original) to 4 (highly original). Inter-rater reliability (IRR = 0.863) and the interclass correlation coefficient (ICC = 0.968) for originality ratings were very good. Next, the originality scores for the two raters were averaged for each idea. The composite score was calculated as the total number of non-identical ideas that were judged to be both malevolent (average malevolence rating ≥ 1) and original (average originality rating ≥ 2). Subsequent statistical analyses focused on the fluency index, malevolence index, and the composite score as the MCT outcome variables.

#### 2.4.2. Statistical Analyses

Data analysis was conducted with IBM SPSS Statistics Version 29.0. From the larger sample of participants (*N* = 276), nine were excluded from further analysis because they did not meet demographic criteria (i.e., males and females between 18–25 years of age). Nine additional participants were excluded due to missing data on variables essential to the current analysis (e.g., the MCBS). One participant was excluded due to poor data quality, such as giving inconsistent answers (e.g., endorsing a behavior then denying that behavior in response to a subsequent question). Two additional participants were excluded from further analysis due to noncompliance on the MCT (*n* = 2) (e.g., responding to the scenario by saying, “I would not seek revenge.”). One participant was excluded due to an MCBS score that was more than three standard deviations higher than the mean. Six participants were identified as MCT outliers due to their high scores; these individuals had values greater than 3 times the interquartile range on measures of MCT fluency index (*n* = 5) or composite score (*n* = 1). The final dataset contained 248 participants.

Next, participant characteristics (demographics, SP-PSP, dark triad personality traits, past MC behaviors), and MCT provocation level and task performance (fluency index, malevolence index, and composite score) were summarized using descriptive statistics. Pearson correlations with list-wise deletion were used to assess the potential inter-relationships between predictor variables (SP-PSP, dark triad personality traits, past MC behaviors, and provocation level), as well as associations between predictor variables and MCT outcomes (fluency index, malevolence index, and composite score). Predictors of MCT outcomes were then examined using a sequential linear regression approach similar to the approach reported by Perchtold-Stefan et al. (2021a). Separate sequential linear regression models were developed for each of the MCT outcomes (fluency index, malevolence index, and composite score) to examine the incremental impact of specific variables or sets of variables while controlling for others, such that each block allowed us to observe changes in the variance explained (∆*R*^2^) as the variables were added in stages. In block 1, age and sex were entered to control for potential age effects related to frontal lobe development from late adolescence to adulthood, as well as sex differences in dark triad traits and/or malevolent creativity (e.g., Casey et al., 2008; Chiorri et al., 2019; Götz et al., 2020; Grijalva et al., 2015; Harris & Reiter-Palmon, 2015; Lee & Dow, 2011). SP-PSP and dark triad personality traits were entered in block 2. To determine if past MC behavior predicted MCT outcomes above and beyond SP-PSP and dark triad traits, past MC behavior was entered alone in block 3. To determine if current provocation level predicted MCT outcomes above and beyond prior variables, provocation level was entered alone in block 4. Finally, we conducted a sensitivity analysis by repeating the sequential linear regressions with the six positive MCT outliers included. For these analyses, the most extreme outliers were winsorized at the 5th and 95th percentiles for MCT fluency index, malevolence index, and composite score. This strategy allowed us to test the robustness of our models with the six positive outliers included without violating the assumptions of linear regression analyses.

All statistical analyses used α = 0.05. Sensitivity power analyses (G*Power 3.1.9.7) (Faul et al., 2007, 2009) determined that our sample size (*N* = 248) was adequately powered (1 − β = 0.80) to detect the small effects that were theoretically expected in bivariate correlations (minimum detectable effect of |*r*| = 0.177) and sequential linear regressions ending with a total of eight predictors across the four blocks (minimum detectable effect of *f*^2^ = 0.063).

## 3. Results

### 3.1. Participant Characteristics

Participant characteristics are shown in Table 1. The final participant sample was composed of 248 undergraduate college students aged 18 to 25 with an average age of approximately 20 years. Most participants were female (82.3%), White (63.7%), and non-Hispanic (56.9%). The proportion of Hispanic students in our study sample (43.1%) was significantly larger than typical representation in the U.S., where approximately 20% of all college students are Hispanic (Hernandez & McElrath, 2023). This is of interest because previous studies have shown variation in the endorsement of dark triad traits in individualistic cultures (e.g., the U.S.) and collectivistic cultures (e.g., Latin America) (Jonason et al., 2013; Jonason et al., 2020; Ramos-Vera et al., 2023; Robertson et al., 2016). Additional research is needed to determine the extent to which such findings might generalize to non-Hispanic versus Hispanic college students in the American Southwest. In the current study population, Hispanic and non-Hispanic students did not differ on their endorsement of narcissism (*p* > .45), psychopathy (*p* > .28), or Machiavellianism (*p* > .14).

Subsequent regression models controlled for age and sex, as age is associated with frontal lobe development and sex is known to influence dark triad traits and/or malevolent creativity (e.g., Casey et al., 2008; Chiorri et al., 2019; Götz et al., 2020; Grijalva et al., 2015; Harris & Reiter-Palmon, 2015; Lee & Dow, 2011). Table 1 also shows the means and standard deviations for SP-PSP, dark triad personality traits, past MC behavior, and MCT provocation level, fluency index, malevolence index, and composite score. Skewness and kurtosis values for the continuous variables were ≤1.5 (age: skewness = 1.2, kurtosis = 1.5; SP-PSP: −0.4, 0.2; Machiavellianism: 0.3, −0.5; narcissism: −0.2, 0.1; psychopathy: 0.5, −0.5; past MC behavior: 0.9, 0.2; provocation level: −0.3, −0.7; MCT Fluency: 0.9, 0.5; MCT Malevolence Index: 0.9, 0.5; Composite Score: 0.9, 0.7).

### 3.2. Correlations

#### 3.2.1. Predictor Variables

Pearson correlations with list-wise deletion were used to assess associations among the predictor variables (summarized in Table 2). SP-PSP was not significantly associated with the other predictor variables, |*r*| < 0.10, *p* > .16. Dark triad personality traits were significantly, positively related to past MC behavior (narcissism: *r*(233) = 0.31, *p* < .001; Machiavellianism: *r*(233) = 0.43, *p* < .001; psychopathy: *r*(233) = 0.33, *p* < .001) and to one another. Narcissism was significantly, positively associated with Machiavellianism, *r*(233) = 0.37, *p* < .001 and psychopathy, *r*(233) = 0.23, *p* < .001. Machiavellianism was significantly, positively associated with psychopathy (*r*(233) = 0.55, *p* < .001); this level of intercorrelation suggests that the Dark Triad Dirty Dozen Questionnaire (Jonason & Webster, 2010) may not have optimally differentiated these constructs in our participant sample. In sequential regression analyses, this intercorrelation may affect the ability of these variables to act as unique predictors of MCT outcomes. Provocation level was significantly associated with past MC behavior, *r*(233) = 0.21, *p* < .001. No other statistically significant associations were noted between predictor variables (|*r*| < 0.11, *p* > .09).

#### 3.2.2. Predictors and Outcome Variables

Pearson correlations with listwise deletion were used to assess associations between predictors and outcome variables (summarized in Table 3). SP-PSP was significantly positively associated with the fluency index, *r*(233) = 0.16, *p* = .01, malevolence index, *r*(233) = 0.15, *p* = .02, and the composite score, *r*(233) = 0.14, *p* = .03. Narcissism was significantly positively associated with the fluency index, *r*(233) = 0.14, *p* = .03, the malevolence index, *r*(233) = 0.16, *p* = .02, and the composite score, *r*(233) = 0.17, *p* = .01. Machiavellianism and psychopathy were not significantly associated with any of the MCT outcome variables in our sample (|*r*| < 0.03, *p* > .63), however, these two dark triad variables were retained in subsequent sequential regression analyses based on their theoretical relevance and to prevent omitted variable bias. Past MC behavior (MCBS score) was significantly positively related to the fluency index, *r*(233) = 0.13, *p* = .04, the malevolence index, *r*(233) = 0.18, *p* = .007, and the composite score, *r*(233) = 0.18, *p* = .005. Provocation level was significantly positively associated with the malevolence index (*r*(233) = 0.17, *p* = .01) and the composite score (*r*(233) = 0.16, *p* = .01), but the positive association with the fluency index only approached statistical significance (*r* = 0.13, *p* = .05).

### 3.3. Sequential Linear Regressions

Separate sequential linear regressions were conducted for each MCT outcome (fluency index, malevolence index, and composite score) to examine the incremental impact of the predictors. For all predictors, the variance inflation factor (VIF) was <1.8, indicating negligible risk of multicollinearity. For each analysis, we assessed the normality of residuals, as well as linearity and homoscedasticity using visual diagnostics. Quartile-Quantile (Q-Q) plots indicated no severe deviations from normality, and plots of residuals against the predicted values indicated that the residuals were randomly dispersed, confirming the assumption of linearity and homoscedasticity. The results of the sequential linear regressions are summarized in Table 4, Table 5 and Table 6 and described in greater detail below. To facilitate comparison with prior studies, we have included partial *η*^2^ values in our descriptions of individual predictors that were statistically significant or showed trends toward significance. For the full models, *f*^2^ are provided.

#### 3.3.1. MCT Fluency Index Regression Models

After controlling for age and sex in block 1, SP-PSP and dark triad traits were entered in block 2. Both SP-PSP (*β* = 0.160, *t* = 2.45, *p* = .02, partial *η*^2^ = 0.03) and narcissism (*β* = 0.156, *t* = 2.22, *p* = .03, partial *η*^2^ = 0.02) were significant predictors in model 2, which accounted for approximately 5% of the variance in the fluency index, *R*^2^ = 0.048, *F*(6, 228) = 1.91, *p* = .08. The addition of past MC behavior (MCBS score) in block 3 did not significantly increase the explained variance (Δ*R*^2^ = 0.009, *p* = .13). SP-PSP (*β* = 0.147, *t* = 2.24, *p* = .03, partial *η*^2^ = 0.02) remained the only significant predictor (although narcissism was a marginally significant predictor, *β* = 0.137, *t* = 1.93, *p* = .06, partial *η*^2^ = 0.02), in model 3, which accounted for approximately 6% of the variance in the fluency index, *R*^2^ = 0.057, *F*(7, 227) = 1.97, *p* = .06. Finally, the addition of provocation level in block 4 did not significantly increase the explained variance (Δ*R*^2^ = 0.008, *p* = .18). SP-PSP (*β* = 0.143, *t* = 2.17, *p* = .03, partial *η*^2^ = 0.02) remained the only significant predictor (although narcissism was a marginally significant predictor, *β* = 0.131, *t* = 1.84, *p* = .07, partial *η*^2^ = 0.02), in model 4, which accounted for approximately 7% of the variance in the fluency index, *R*^2^ = 0.065, *F*(8, 226) = 1.96, *p* = .05, representing a small effect size (*f*^2^ = 0.07).

#### 3.3.2. MCT Malevolence Index Regression Models

After controlling for age and sex in block 1, SP-PSP and dark triad traits were entered in block 2. Both SP-PSP (*β* = 0.152, *t* = 2.32, *p* = .02, partial *η*^2^ = 0.02) and narcissism (*β* = 0.171, *t* = 2.44, *p* = .02, partial *η*^2^ = 0.03) were significant predictors in model 2, which accounted for 5% of the variance in the malevolence index, *R*^2^ = 0.050, *F*(6, 228) = 2.02, *p* = .06. The addition of past MC behavior (MCBS score) in block 3 significantly increased the explained variance (Δ*R*^2^ = 0.021, *p* = .03). SP-PSP (*β* = 0.133, *t* = 2.03, *p* = .04, partial *η*^2^ = 0.02), narcissism (*β* = 0.144, *t* = 2.04, *p* = .04, partial *η*^2^ = 0.02), and past MC behavior (*β* = 0.164, *t* = 2.24, *p* = .03, partial *η*^2^ = 0.03) were significant predictors in model 3, which accounted for approximately 7% of the variance in the malevolence index (*R*^2^ = 0.071, *F*(7, 227) = 2.48, *p* = .02). Finally, the addition of provocation level in block 4 did not significantly increase the explained variance (Δ*R*^2^ = 0.014, *p* = .07). The overall model remained significant (*R*^2^ = 0.084, *F*(8, 226) = 2.61, *p* = .01) and accounted for approximately 8% of the variance in the malevolence index, representing a small effect size (*f*^2^ = 0.09). However, the individual predictive values of SP-PSP (*β* = 0.127, *t* = 1.95, *p* = .05, partial *η*^2^ = 0.02), narcissism (*β* = 0.135, *t* = 1.92, *p* = .06, partial *η*^2^ = 0.02), past MC behavior (*β* = 0.139, *t* = 1.88, *p* = .06, partial *η*^2^ = 0.02), and provocation level (*β* = 0.120, *t* = 1.83, *p* = .07, partial *η*^2^ = 0.02) merely approached significance in model 4. Because VIFs for predictors in this model are <1.8, it is unlikely that this instance of a significant overall model with non-significant individual predictors is due to multicollinearity. Rather, it is more likely due to a suppression effect in which the combined explanatory power of the model is greater than the sum of the model’s individual parts.

#### 3.3.3. MCT Composite Score Regression Models

After controlling for age and sex in block 1, SP-PSP and dark triad traits were entered in block 2. SP-PSP (*β* = 0.129, *t* = 1.98, *p* = .049, partial *η*^2^ = 0.02) and narcissism (*β* = 0.189, *t* = 2.71, *p* = .007, partial *η*^2^ = 0.03) were significant predictors in model 2, which accounted for approximately 5% of the variance in the composite score, *R*^2^ = 0.053, *F*(6, 228) = 2.12, *p* = .05. The addition of past MC behavior (MCBS score) in block 3 significantly increased the explained variance (Δ*R*^2^ = 0.026, *p* = .01). Narcissism (*β* = 0.158, *t* = 2.25, *p* = .03, partial *η*^2^ = 0.02) and past MC behavior (*β* = 0.186, *t* = 2.55, *p* = .01, partial *η*^2^ = 0.03) were significant predictors in model 3, which accounted for approximately 8% of the variance in the composite score, *R*^2^ = 0.079, *F*(7, 227) = 2.79, *p* = .008. Finally, the addition of provocation level in block 4 did not significantly increase the explained variance (Δ*R*^2^ = 0.012, *p* = .08). Narcissism (*β* = 0.150, *t* = 2.14, *p* = .03, partial *η*^2^ = 0.02) and past MC behavior (*β* = 0.162, *t* = 2.20, *p* = .03, partial *η*^2^ = 0.02) were significant predictors in model 4, which accounted for approximately 9% of the variance in the composite score, *R*^2^ = 0.091, *F*(8, 226) = 2.84, *p* = .005, representing a small effect size (*f*^2^ = 0.10).

### 3.4. Sensitivity Analyses

#### 3.4.1. Examining MCT Noncompliance

Two participants were excluded from statistical analyses for MCT non-compliance. To evaluate potential non-response bias, we compared the non-responder mean on predictor variables to that of the analyzed participant sample; because there were only two participants who were noncompliant, it was not possible to make statistical comparisons. For SP-PSP, dark triad, and MCBS scores, the non-responder mean fell within the normal spread of scores (e.g., within 3 *SD* of the mean for the analyzed participant sample). Compared to the mean for the analyzed participant sample, the non-responder mean was higher for SP-PSP (non-responder *M* = 4.50; analyzed sample *M* = 3.53), lower for psychopathy (non-responder *M* = 1.38; analyzed sample *M* = 2.05) and Machiavellianism (non-responder *M* = 1.25; analyzed sample *M* = 2.33), higher for narcissism (non-responder *M* = 3.50; analyzed sample *M* = 3.06), and lower for MCBS (non-responder *M* = 1.16; analyzed sample *M* = 1.59). For provocation, one participant did not respond to the question at all, while the other participant gave a score of “1”, which was lower than the analyzed participant sample mean (*M* = 4.08) but within the normal spread of scores. While the non-responders were comparable to the analyzed sample in terms of the predictor variables, their non-participation may still stem from other, unmeasured systematic characteristics.

#### 3.4.2. Examining the Robustness of Regression Models

Sensitivity analyses were conducted by repeating the sequential linear regressions with the six positive outliers on the MCT variables included. For these analyses, the most extreme cases were winsorized at the 5th and 95th percentiles, a strategy that allowed us to test the robustness of our models without violating the assumptions of linear regression.

#### 3.4.3. MCT Fluency Index

As in previous analyses, both SP-PSP (*β* = 0.128, *t* = 1.98, *p* = .049, partial *η*^2^ = 0.02) and narcissism (*β* = 0.164, *t* = 2.36, *p* = .02, partial *η*^2^ = 0.02) were significant predictors in model 2, which accounted for approximately 4% of the variance in the fluency index, *R*^2^ = 0.044, *F*(6, 234) = 1.80, *p* = .10. Also similar to previous analyses, the addition of past MC behavior (MCBS score) in block 3 did not significantly increase the explained variance (Δ*R*^2^ = 0.013, *p* = .07). However, contrary to previous analyses, narcissism (*β* = 0.140, *t* = 1.99, *p* = .048, partial *η*^2^ = 0.02) was the only significant predictor (although SP-PSP, *β* = 0.115, *t* = 1.77, *p* = .08, partial *η*^2^ = 0.01, and past MC behavior, *β* = 0.132, *t* = 1.81, *p* = .07, partial *η*^2^ = 0.02, approached significance) in model 3, which accounted for approximately 6% of the variance in the fluency index, *R*^2^ = 0.057, *F*(7, 233) = 2.03, *p* = .05. Finally, as in previous analyses, the addition of provocation level in block 4 did not significantly increase the explained variance (Δ*R*^2^ = 0.007, *p* = .18). The overall model remained significant (*R*^2^ = 0.065, *F*(8, 232) = 2.01, *p* = .047) in model 4 and accounted for approximately 7% of the variance in the fluency index, which was similar to previous analyses, representing a small effect size (*f*^2^ = 0.07). However, in contrast to previous analyses, the individual predictive values of narcissism (*β* = 0.132, *t* = 1.88, *p* = .06, partial *η*^2^ = 0.02) and SP-PSP (*β* = 0.113, *t* = 1.74, *p* = .08, partial *η*^2^ = 0.01) merely approached significance in model 4.

To further investigate this issue and to determine whether SP-PSP was merely a less effective predictor of MCT fluency for higher performers, we examined differences in beta metrics (DFBETAs) for SP-PSP scores in the MCT fluency sensitivity analysis. We found that only one of the six individuals who were positive MCT outliers also had an extreme DFBETA value for SP-PSP (greater than three times the interquartile range). This suggested that the inclusion of that participant, who had high MCT fluency paired with a very low SP-PSP score, significantly reduced the predictive utility of SP-PSP for MCT fluency. When the sensitivity analysis was repeated with that participant removed, SP-PSP was the only significant individual predictor of MCT fluency (*β* = 0.143, *t* = 2.20, *p* = .03, partial *η*^2^ = 0.02) in model 4 (*R*^2^ = 0.067, *F*(8, 231) = 2.03, *p* = .04), which accounted for approximately 7% of the variance in the fluency index, which was similar to previous analyses, representing a small effect size (*f*^2^ = 0.07).

#### 3.4.4. MCT Malevolence Index

In contrast to previous analyses, narcissism was the only significant predictor (*β* = 0.179, *t* = 2.58, *p* = .01, partial *η*^2^ = 0.03) (although SP-PSP approached significance, *β* = 0.120, *t* = 1.86, *p* = .06, partial *η*^2^ = 0.01) in model 2, which accounted for approximately 5% of the variance in the malevolence index, *R*^2^ = 0.049, *F*(6, 234) = 1.99, *p* = .07. As in previous analyses, the addition of past MC behavior (MCBS score) in block 3 significantly increased the explained variance (Δ*R*^2^ = 0.025, *p* = .01). Narcissism (*β* = 0.146, *t* = 2.09, *p* = .04, partial *η*^2^ = 0.02), and past MC behavior (*β* = 0.183, *t* = 2.52, *p* = .01, partial *η*^2^ = 0.03) were significant predictors in model 3, which accounted for approximately 7% of the variance in the malevolence index, *R*^2^ = 0.074, *F*(7, 233) = 2.65, *p* = .01. Finally, as in previous analyses, the addition of provocation level in block 4 did not significantly increase the explained variance (Δ*R*^2^ = 0.013, *p* = .08). Past MC behavior (*β* = 0.158, *t* = 2.15, *p* = .03, partial *η*^2^ = 0.02) was the only significant predictor (although narcissism approached significance, *β* = 0.136, *t* = 1.95, *p* = .05, partial *η*^2^ = 0.02) in model 4, which accounted for approximately 9% of the variance in the malevolence index (*R*^2^ = 0.086, *F*(8, 232) = 2.74, *p* = .007), representing a small effect size (*f*^2^ = 0.09) similar to the previous analyses.

#### 3.4.5. MCT Composite Score

In contrast to previous analyses, narcissism (*β* = 0.185, *t* = 2.66, *p* = .008, partial *η*^2^ = 0.03) was the only significant predictor in model 2, which accounted for approximately 4% of the variance in the composite score, *R*^2^ = 0.042, *F*(6, 234) = 1.70, *p* = .12. As in previous analyses, the addition of past MC behavior (MCBS score) in block 3 significantly increased the explained variance (Δ*R*^2^ = 0.028, *p* = .009). Narcissism (*β* = 0.150, *t* = 2.15, *p* = .03, partial *η*^2^ = 0.02) and past MC behavior (*β* = 0.192, *t* = 2.65, *p* = .009, partial *η*^2^ = 0.03) were significant predictors in model 3, which accounted for 7% of the variance in the composite score, *R*^2^ = 0.070, *F*(7, 233) = 2.50, *p* = .02. Finally, as in previous analyses, the addition of provocation level in block 4 did not significantly increase the explained variance (Δ*R*^2^ = 0.011, *p* = .09). Narcissism (*β* = 0.141, *t* = 2.02, *p* = .045, partial *η*^2^ = 0.02) and past MC behavior (*β* = 0.168, *t* = 2.28, *p* = .02, partial *η*^2^ = 0.02) were significant predictors (although provocation level approached significance, *β* = 0.111, *t* = 1.70, *p* = .09, partial *η*^2^ = 0.01) in model 4, which accounted for approximately 8% of the variance in the composite score (*R*^2^ = 0.081, *F*(8, 232) = 2.56, *p* = .01), representing a slightly smaller effect size (*f*^2^ = 0.09) than the previous analyses.

## 4. Discussion

The current study was designed to test how self-reported past MC behavior may influence malevolently creative ideation beyond the individual-level factors of self-perception of problem-solving potential (SP-PSP) and the dark triad personality traits, which align with the individual-level mechanisms (i.e., the “M”) in the AMORAL model. The potential influence of provocation level, which aligns with the environment level (i.e., environmental operants, the “O” in the AMORAL model), on MCT was also examined to determine whether it also accounted for unique variance beyond the aforementioned factors, based on previous findings of malevolently creative ideation in response to provocation (e.g., Perchtold-Stefan et al., 2021a). Malevolently creative ideation was measured by the MCT fluency index, malevolence index, and composite score. All regression analyses were controlled for age and sex, as these factors are associated with frontal lobe development and measures of dark triad traits and malevolent creativity (e.g., Casey et al., 2008; Chiorri et al., 2019; Götz et al., 2020; Grijalva et al., 2015; Harris & Reiter-Palmon, 2015; Lee & Dow, 2011). The hypotheses were largely supported. The strengths and directions of associations between our individual predictors and MCT outcome variables were consistent with our predictions and aligned with previous studies that showed small to medium effects (Wahbeh et al., 2024; Z. Gao et al., 2022; Hao et al., 2020; Perchtold-Stefan et al., 2021a). In regression analyses, the strongest predictor of fluency index was SP-PSP. For the malevolence index, the addition of past MC behavior explained a significant amount of variance beyond SP-PSP and the dark triad. For the composite score, both narcissism and past MC behavior emerged as predictors of the composite score, with past MC behavior showing significant predictive power independent of the dark triad. Finally, although provocation level was significantly correlated with both the malevolence index and the composite score, provocation level was not a significant predictor of MCT outcomes in regression analyses. Taken together, these findings indicate that past MC behaviors uniquely contribute to malevolent creativity ideation beyond personality and SP-PSP. The significant but relatively modest contributions (7% to 9% of the variance across the three models) of these factors to malevolently creative ideation are consistent with the AMORAL model (Kapoor & Kaufman, 2022a), which posits that a multitude of factors underlie MC behaviors. The current results highlight that past MC behaviors add predictive power beyond the individual-level mechanisms of SP-PSP and narcissism and the environment-level factor of current provocation level, supporting the notion that an array of antecedents, environmental operants, and individual mechanisms all contribute to the emergence of MC behaviors.

However, it is more difficult to directly compare the predictive strengths of our final models (i.e., 7% to 9% of the variance) to the existing literature, given the unique combinations of AMORAL-aligned variables that have been included as predictors across studies of MC ideation. Further, most studies using the MCT or related tasks in college student samples were conducted in cultures different than our own (e.g., Austria and China); as mentioned previously, cultural differences have been implicated as predictors of malevolent creativity (Jonason et al., 2013; Jonason et al., 2020; Ramos-Vera et al., 2023; Robertson et al., 2016). For similar studies, a range of *R*^2^ values have been reported for the final models. For example, Perchtold-Stefan et al. (2021a) reported an *R*^2^ = 0.33 for their final model, which included gender, conventional creative ideation, maladaptive personality, and state anger as predictors of the MCT composite score. However, other studies are more in line with the predictive strengths reported here. For example, Z. Gao et al. (2022) noted that the simultaneous addition of MC behaviors, dark triad traits, and trait aggression in the final block a of a sequential regression accounting for age, sex, and creative behavioral tendencies (measured via the Runco Ideational Behavior Scale; Runco et al., 2016), increased the explained variance in MCT originality by 7%, with MC behaviors remaining as the only significant predictor in the final model. Likewise, Hao et al. (2016) report that MCBS accounted for 7% of the variance in MC task performance. Additional cross-cultural comparisons are needed to fully understand the relationship between AMORAL-aligned variables and malevolently creative ideation in response to provocation.

As predicted in Hypothesis 1, people who perceived themselves to have greater creative potential in everyday problem-solving generated a larger number of ideas. The fluency index focused on the total count of task-relevant ideas without regard to quality or originality. It is not surprising that SP-PSP remained the key predictor of fluency after accounting for all other factors, as previous studies have shown positive associations between malevolent creativity and conventional creative cognition, suggesting that malevolent creativity may involve similar cognitive processes that allow for fluent generation of ideas (Perchtold-Stefan et al., 2021a). Previous work has demonstrated that self-reported creativity often predicts divergent thinking fluency (e.g., Callan et al., 2021; Lebuda et al., 2025), suggesting that in the current experiment, fluency may be attributable to divergent thinking ability, as participants were explicitly asked to generate as many original ideas as possible. One possibility is that individuals with higher fluency indices may have engaged in more associative thinking and/or were able to more easily connect to weakly associated ideas through a broader associative network (Mednick, 1962). Another possibility is that they may have been better at analogically transferring the conceptual structure of a previously encountered episode to the currently presented scenario, resulting in additional avenues for creative ideation (Schooler & Melcher, 1995). Additional research relating performance on divergent thinking or other standard creativity tasks rather than a single self-reported measure of creative potential to the MCT fluency index would provide more direct support for these possibilities.

The malevolence index refined the analysis by focusing solely on ideas that were judged by the two independent raters to be at least slightly malevolent according to the rubric published by Perchtold-Stefan et al. (2021a). For the malevolence index, not only SP-PSP, but also narcissism and past MC behavior emerged as significant predictors, with past MC behavior contributing unique predictive power to the model above and beyond SP-PSP and dark-triad traits. The overall model remained significant after the addition of provocation level as a predictor, although the contributions of individual predictors only approached significance. Hypothesis 2 predicted that individuals who score high in narcissism would be more likely than the other dark triad types to exhibit better performance on the malevolence index. This prediction was supported by previous research, which suggests that narcissists are ego-driven and thus may be particularly reactive to ego threats and social slights (Lambe et al., 2018), such as the one illustrated in the Neighbor/Money scenario. In contrast, individuals who score highly on psychopathy tend to be more proactive in their antisocial behavior, and individuals with Machiavellian traits are described as being manipulative or scheming, and more apt to focus on intricate, longer-range plans for revenge, a style that may not translate as well to the current task which prioritizes the generation of many short ideas for revenge (Paulhus & Williams, 2002). Studies examining how each of the dark triad traits relates to different MC scenarios that vary across time scales (e.g., require short- or long-term revenge schemes) and ego involvement are needed to provide additional support for this hypothesis.

Hypothesis 3 predicted that past MC behavior would be positively associated with the MCT malevolence index and composite index and would account for unique variance above and beyond that of dark triad traits. The composite score provided a measure of malevolent creativity that conforms to the standard definition of creativity, in that it reflected the number of ideas that were judged to be both malevolent and original. This finding is interesting in light of past criticisms of the MCBS, namely that it measures malicious or antagonistic intent rather than creativity, which would require originality behind the actions (Waldie et al., 2021). Perchtold-Stefan et al. (2021a) found that MCBS scores were correlated with MCT performance. Our study moves this a step further by showing that MCBS scores contributed new information to the model predicting malevolent creativity composite scores, rather than merely duplicating what other predictors (age, sex, SP-PSP, dark triad personality) already explain. For the MCT composite index, past malevolent behavior and narcissism remained significant predictors even after the addition of provocation level to the model.

In their study of Romanian prisoners, Szabó et al. (2022) found that more experienced criminals exhibited better performance on the MCT. Both of these findings are consistent with the notion that practice may make perfect, suggesting that when provoked, individuals with a particular set of antecedents and individual factors (the accelerators and handbrakes of the AMORAL model; Kapoor & Kaufman, 2022a) may use their creativity as a tool for revenge (e.g., dark decision-making; Runco, 2010), although additional research establishing a causal connection between past MC behavior and MCT performance is necessary to strengthen this claim. As noted earlier, this may be particularly true of narcissists who might use malevolently creative behaviors to alleviate the negative emotions associated with narcissistic injury and to restore a sense of control (e.g., Lambe et al., 2018). Consistent with social learning theory (Akers, 2001), malevolently creative behavior may develop over time through the achievement of desirable outcomes when malevolently creative behavior is expressed. Eventually, MC ideation and behavior could become a component of a reinforcing behavioral feedback loop, an allostatic regulatory system, or a vicious cycle in which hostile actions are reinforced (McClelland, 2010; Perchtold-Stefan et al., 2021b), resulting in a habitual or automatic cognitive strategy (Graybiel, 2008). Although this possibility cannot be addressed within the current dataset, the small yet statistically significant amount of unique variance accounted for by past MC behaviors in our college student sample suggests that the contribution of reinforcement learning to malevolently creative ideation deserves further attention. This reinforces the importance of the comprehensive interdisciplinary approach of the AMORAL model, which accounts for malleable factors such as socio-emotional skills and the intersection of creativity and moral reasoning (Kapoor & Kaufman, 2022b, 2023; Zhao et al., 2022).

Hypothesis 4 predicted that provocation level would be positively associated with MCT outcomes, particularly the malevolence index and the composite score; these relationships were confirmed in correlational analyses, which showed small effects. Given the importance of environment-level predictors as potential amplifiers of malevolent creativity in the AMORAL model, it may be surprising that provocation level did not uniquely predict MCT outcomes. One possibility is that other environmental operants, which were not measured or manipulated here, might have shown stronger predictive relationships with MCT outcomes. Nonetheless, it is worth noting that the characteristics of the provocation data in our study (*M* = 4.08, *SD* = 1.45) were within range of those reported by Perchtold-Stefan et al. (2021a) (*M* = 4.37, *SD* = 1.02), although those authors did not examine potential associations between provocation level and MCT outcomes. One potential explanation for our finding is that our study of college students focused on a relatively homogenous population of emerging adults with limited life experiences, which may have restricted their “aggressive expertise” (as described by Perchtold-Stefan et al., 2025), and thus, the extent to which provocation elicited malevolently creative ideas. In a broader participant sample drawn from the general public with a greater range of life experiences, it is possible that provocation level might be a stronger predictor of MC ideation.

Finally, we tested the robustness of our models by conducting sensitivity analyses, in which the sequential linear regression models were repeated with the six positive outliers on MCT outcome variables included. In general, the results of these analyses supported the robustness of our previous findings. As in the analysis with these six outliers removed, the proportion of variance explained by our predictors in the final models of the sensitivity analysis ranged from 7% to 9%, indicating that the inclusion of individuals with higher MCT outcome scores still resulted in small effects.

## 5. Limitations, Implications, and Future Directions

This was a cross-sectional survey study, and, as such, findings focused on associations rather than cause and effect. Extension of this work in longitudinal models of malevolently creative behavior and ideation is needed to support potential mechanistic explanations for these relationships.

We examined the three MCT outcomes in separate sequential regression models; this was justified due to the nature of the differences between these variables. Fluency measured raw productivity, whereas the malevolence index focused exclusively on malevolence, and the composite score added the element of originality to more adequately reflect true malevolent creativity. However, this analysis strategy may have increased the probability of Type 1 errors. Therefore, rather than relying solely on probability values, we have also considered the magnitude of the effects by emphasizing the percentage of variance explained and the effect sizes, as these remain accurate regardless of the number of analyses conducted. For each MCT outcome, the final model accounted for only 7% to 9% of the total variance, corresponding to a small effect size. While this is a small number in absolute terms, the variables queried here are just a subset of the large number of factors theorized to contribute to malevolent creativity (Kapoor & Kaufman, 2022a). These other factors were not included in the current study for practical reasons but remain to be addressed in future research.

This study included a within-subjects experimental element, in that participants were provoked by a stimulus (Neighbor/Money Scenario) and then responded in real time, albeit without a time constraint for idea generation. While MCT data were collected without the control afforded by lab-based tasks, our methods conform to those employed in the problem-based approach of Harris and Reiter-Palmon (2015). Further, Dumas and Strickland (2018) note that the collection of malevolently creative ideas outside the laboratory via online methods may be beneficial, as it allows for potentially advantageous privacy and flexibility, important considerations given the social desirability limitations associated with the study of malevolent creativity. However, it is also important to acknowledge that our participants were compensated via course credit. While this is a standard approach in psychological research, it is a potential source of demand characteristics that might alter natural behavior, especially for self-report measures of socially undesirable actions.

For conciseness, we used a single-item measure of self-perception of creative potential specifically related to everyday problem-solving rather than using an entire validated questionnaire. However, the use of a single-item measure may have led our regression models to underestimate the true effect of the SP-PSP. Further, it is subjective and thus does not fully capture creative ability or capacity. Additionally, the untimed, open response format of the MCT may have favored verbally fluent participants, inflating both fluency and malevolence indices for these individuals, irrespective of the predictor variables of interest. Future studies that include a traditional, objective test of general creative ideation and that control for verbal fluency are needed to further substantiate our findings.

Because presentation of the MCBS and SP-PSP preceded the MCT, it is possible that the presentation of the general questions contained in the MCBS may have acted as a prime for MCT performance. However, because the SP-PSP was administered between the MCBS and the MCT, this intervening item may have decreased the potential priming of MCT by MCBS. Counterbalancing the administration order of these items in future studies may help to clarify the role of MCBS as a stable trait predictor versus a contextual prime.

Another limitation is that the 12-item Dirty Dozen (Jonason & Webster, 2010), which was used to measure dark triad traits in the current study, may provide lower discriminant validity compared to longer dark triad measures (Knitter et al., 2025). Of particular relevance to the results of the current study, the Dirty Dozen may inadequately capture and differentiate between the covert/vulnerable and overt/grandiose features of narcissism (Kajonius et al., 2016). While both types may be triggered by ego-threat provocation, they differ in emotional and social-cognitive responses (Hart et al., 2017). Overt/grandiose narcissists tend to be more “thick-skinned”, assertive, and attentive to self-image, whereas covert/vulnerable narcissists may be hypersensitive and “thin-skinned”, exhibiting extreme sensitivity to slights (Gabbard, 1989; Levy, 2012; Maples et al., 2025). More recent work (Maples et al., 2025) has further explored these subtypes of narcissism in relation to antagonism and aggression, which are particularly relevant to MCT performance. A full exploration of these factors was beyond the scope of the current study, but future research should consider the different subtypes of narcissistic personality and their potential roles in malevolent creativity.

Finally, the current sample was limited to college students from a single university and was primarily female (82%). Dark triad traits tend to be stronger in males than in females and in younger compared with older adults (e.g., Casey et al., 2008; Chiorri et al., 2019; Götz et al., 2020; Grijalva et al., 2015; Harris & Reiter-Palmon, 2015; Lee & Dow, 2011), and there may also be sex differences in malevolently creative ideation (Dumas & Strickland, 2018), although this finding is less consistent (e.g., Al-Mahdawi et al., 2022; Perchtold-Stefan et al., 2023). In the current study, both sex and age were accounted for in the linear regression models, but the lack of sex, age, and geographic diversity of the sample limits the generalizability of these findings to individuals who are not young-adult females in the Southcentral United States.

Despite these limitations, the results of the current study support the AMORAL model (Kapoor & Kaufman, 2022a, 2023) and suggest directions for future studies to explore how influences beyond individual mechanisms and provocation level shape malevolently creative ideation.

## 6. Conclusions

This research highlights that understanding malevolence requires a careful look at an individual’s personality, lived experiences, and surrounding context. The results of this study contribute to a growing body of research on malevolent creativity by showing how SP-PSP, past MC behaviors, dark triad traits, and current provocation level individually contribute to the generation of malevolently creative ideas for revenge. SP-PSP appears to be more strongly related to the general ability to generate ideas, narcissism is related to the generation of specifically malevolent ideas, and past MC behaviors uniquely predicted the composite score (a true measure of malevolent creativity that includes originality). However, replication beyond a single-university sample is necessary, given the modest significance levels achieved here and the impact that outliers had on significance levels, as highlighted by the sensitivity analyses. One possibility that remains to be tested is that past MC behaviors may influence future malevolent creativity through reinforcement learning and habit formation, although other underlying dynamics are likely to also contribute. Additional longitudinal research is needed to fully understand how past MC behaviors contribute to future MC behaviors. While there are undoubtedly other factors at play, the current results help to elucidate the roles of some of the most popular explanations for malevolently creative ideation.

## Figures and Tables

**Table 1:** Participant characteristics and Malevolent Creativity Task performance (*n* = 248).

Characteristics/MCT Performance	Mean (SD) or % (*n*)
Age	20.0 (1.5)
Sex	
Female	82.3% (204)
Male	17.7% (44)
Ethnicity	
Hispanic	43.1% (107)
Non-Hispanic	56.9% (141)
Race	
Asian	5.2% (13)
Black or African American	14.9% (37)
White	63.7% (158)
Multiracial or Other	14.9% (37)
Declined to Answer	1.3% (3)
SP-PSP	3.53 (0.85)
Dark triad personality traits	
Machiavellianism	2.33 (0.94)
Psychopathy	2.05 (0.84)
Narcissism	3.06 (0.82)
Past MC behavior	1.59 (0.50)
Malevolent Creativity Task	
Provocation level	4.08 (1.45)
Fluency index	2.17 (1.18)
Malevolence index	1.95 (1.27)
Composite score	1.17 (1.00)

Note. MCT = Malevolent Creativity Task; SP-PSP = self-perception of problem-solving potential.

**Table 2:** Associations between predictor variables.

Predictor Variables	1	2	3	4	5	6
1. SP-PSP	-					
2. Machiavellianism	−0.039	-				
3. Psychopathy	−0.093	0.546 ***	-			
4. Narcissism	0.025	0.373 ***	0.232 ***	-		
5. Past MC behavior	0.090	0.429 ***	0.327 ***	0.311 ***	-	
6. Provocation level	0.058	0.043	0.095	0.111	0.214 ***	-

Note. *** *p* < .001; SP-PSP = self-perception of problem-solving potential; MC = malevolently creative.

**Table 3:** Associations between predictors and outcome variables.

Predictor Variables	Fluency Index	Malevolence Index	Composite Score
SP-PSP	0.161 *	0.149 *	0.141 *
Machiavellianism	0.020	0.022	−0.002
Psychopathy	0.015	0.003	−0.031
Narcissism	0.144 *	0.156 *	0.169 *
Past MC behavior	0.134 *	0.177 **	0.181 **
Provocation level	0.128	0.166 *	0.160 *

Note. * *p* < .05; ** *p* ≤ .01; SP-PSP = self-perception of problem-solving potential; MC = malevolently creative.

**Table 4:** Sequential regression model predicting Malevolent Creativity Task fluency index.

	Model 1					Model 2					Model 3					Model 4				
	*B*	*SE*	β	*t*	*p*	*B*	*SE*	β	*t*	*p*	*B*	*SE*	β	*t*	*p*	*B*	*SE*	β	*t*	*p*
Block 1																				
Age	0.016	0.051	0.021	0.31	.76	0.011	0.051	0.015	0.223	.82	0.016	0.051	0.021	0.313	.75	0.020	0.051	0.026	0.394	.69
Gender	−0.009	0.203	−0.003	−0.04	.97	−0.118	0.205	−0.039	−0.572	.57	−0.110	0.205	−0.036	−0.538	.59	−0.107	0.205	−0.035	−0.524	.60
*R* ^2^	0.000																			
*F*	0.048																			
Block 2																				
SP-PSP						0.226	0.092	0.160	2.45	.02	0.208	0.093	0.147	2.24	.03	0.202	0.093	0.143	2.17	.03
Narciss.						0.224	0.101	0.156	2.22	.02	0.197	0.102	0.137	1.93	.06	0.188	0.102	0.131	1.83	.07
Psychop.						0.034	0.113	0.023	0.295	.77	0.010	0.114	0.007	0.087	.93	−0.001	0.114	−0.001	−0.009	.99
Machiav.						−0.059	0.104	−0.047	−0.567	.57	−0.100	0.108	−0.079	−0.929	.35	−0.086	0.108	−0.068	−0.801	.42
*R* ^2^						0.048														
*F*						1.91														
∆*R*^2^						0.048 *														
∆*F*						2.85 *														
Block 3																				
Past MC											0.267	0.178	0.111	1.50	.13	0.222	0.181	0.092	1.23	.22
*R* ^2^											0.057									
*F*											1.97									
∆*R*^2^											0.009									
∆*F*											2.26									
Block 4																				
Provoc.																0.074	0.055	0.090	1.35	.18
*R* ^2^																0.065 *				
*F*																1.96 *				
∆*R*^2^																0.008				
∆*F*																1.83				

Note. * *p* ≤ .05; SP-PSP = self-perception of problem-solving potential; Narciss. = narcissism; Psychop. = psychopathy; Machiav. = Machiavellianism; Past MC = past malevolently creative (MC) behavior; Provoc. = provocation level.

**Table 5:** Sequential regression model predicting the Malevolent Creativity Task malevolence index.

	Model 1					Model 2					Model 3					Model 4				
	*B*	*SE*	β	*t*	*p*	*B*	*SE*	β	*t*	*p*	*B*	*SE*	β	*t*	*p*	*B*	*SE*	β	*t*	*p*
Block 1																				
Age	−0.011	0.055	−0.014	−0.206	.84	−0.015	0.055	−0.018	−0.278	.78	−0.008	0.054	−0.009	−0.145	.88	−0.002	0.054	−0.002	−0.036	.97
Gender	−0.085	0.219	−0.026	−0.390	.70	−0.199	0.222	−0.061	−0.900	.37	−0.188	0.220	−0.057	−0.855	.39	−0.183	0.219	−0.056	−0.839	.40
*R* ^2^	0.001																			
*F*	0.113																			
Block 2																				
SP-PSP						0.231	0.100	0.152	2.32	.02	0.202	0.100	0.133	2.03	.04	0.194	0.099	0.127	1.95	.05
Narciss.						0.266	0.109	0.171	2.44	.02	0.223	0.110	0.144	2.04	.04	0.210	0.109	0.135	1.92	.06
Psychop.						0.018	0.122	0.012	0.149	.88	−0.019	0.122	−0.013	−0.158	.87	−0.035	0.122	−0.023	−0.289	.77
Machiav.						−0.062	0.113	−0.045	−0.549	.58	−0.127	0.115	−0.092	−1.1	.27	−0.107	0.115	−0.078	−0.931	.35
*R* ^2^						0.050														
*F*						2.02														
∆*R*^2^						0.049 *														
∆*F*						2.97 *														
Block 3																				
Past MC											0.427	0.191	0.164	2.24	.03	0.362	0.193	0.139	1.88	.06
*R* ^2^											0.071 *									
*F*											2.48 *									
∆*R*^2^											0.021 *									
∆*F*											5.02 *									
Block 4																				
Provoc.																0.107	0.059	0.120	1.83	.07
*R* ^2^																0.084 **				
*F*																2.61 **				
∆*R*^2^																0.014				
∆*F*																3.34				

Note. * *p* ≤ .05; ** *p* ≤ .01; SP-PSP = self-perception of problem-solving potential; Narciss. = narcissism; Psychop. = psychopathy; Machiav. = Machiavellianism; Past MC = past malevolently creative (MC) behavior; Provoc. = provocation level.

**Table 6:** Sequential regression model predicting Malevolent Creativity Task composite score.

	Model 1					Model 2					Model 3					Model 4				
	*B*	*SE*	β	*t*	*p*	*B*	*SE*	β	*t*	*p*	*B*	*SE*	β	*t*	*p*	*B*	*SE*	β	*t*	*p*
Block 1																				
Age	−0.010	0.044	−0.015	−0.224	.82	−0.010	0.043	−0.015	−0.232	.82	−0.003	0.043	−0.005	−0.081	.94	0.001	0.043	0.001	0.023	.98
Gender	0.129	0.173	0.050	0.746	.46	0.058	0.175	0.022	0.331	.74	0.068	0.173	0.026	0.394	.69	0.072	0.173	0.028	0.416	.68
*R* ^2^	0.002																			
*F*	0.283																			
Block 2																				
SP-PSP						0.156	0.079	0.129	1.98	.049	0.130	0.079	0.108	1.65	.10	0.124	0.078	0.103	1.58	.12
Narciss.						0.233	0.086	0.189	2.71	.007	0.195	0.086	0.158	2.25	.03	0.185	0.086	0.150	2.14	.03
Psychop.						−0.049	0.097	−0.040	−0.507	.61	−0.083	0.097	−0.068	−0.858	.39	−0.095	0.096	−0.077	−0.983	.33
Machiav.						−0.049	0.089	−0.045	−0.555	.58	−0.108	0.091	−0.099	−1.19	.24	−0.093	0.091	−0.086	−1.03	.31
*R* ^2^						0.053 *														
*F*						2.12 *														
∆*R*^2^						0.050 *														
∆*F*						3.03 *														
Block 3																				
Past MC											0.384	0.150	0.186	2.55	.01	0.335	0.152	0.162	2.20	.03
*R* ^2^											0.079 **									
*F*											2.79 **									
∆*R*^2^											0.026 **									
∆*F*											6.51 **									
Block 4																				
Provoc.																0.080	0.046	0.114	1.74	.08
*R* ^2^																0.091 ***				
*F*																2.84 ***				
∆*R*^2^																0.012				
∆*F*																3.02				

Note. * *p* ≤ .05; ** *p* ≤ .01; *** *p* ≤ .005; SP-PSP = self-perception of problem-solving potential; Narciss. = narcissism; Psychop. = psychopathy; Machiav. = Machiavellianism; Past MC = past malevolently creative (MC) behavior; Provoc. = provocation level.

## Data Availability

Upon publication of the manuscript, the original data presented in the study will be openly available in the Texas Data Repository at (https://dataverse.tdl.org/).

## Abbreviations

The following abbreviations are used in this manuscript:

RAT	Remote associates task
MCT	Malevolent creativity task
SP-PSP	Self-perception of problem-solving potential
MC	Malevolently creative
MCBS	Malevolent Creativity Behavior Scale
ICC	Interclass correlation coefficient
IRR	Inter-rater reliability
